# Progress in Surgery

**Published:** 1897-05-15

**Authors:** 


### Progress in Surgery.

### SURGERY OF THE SPINE.

**Lumbar Puncture.**—Reference has frequently been made in The Hospital[1] to the progress which this diagnostic measure has made within recent months. A complete *résumé* of the subject is given by Miles,[2] in which the results of between five and six hundred punctures is summarised. After mentioning the rationale and technique of the proceeding he points out certain difficulties which different operators have met with in the operation. These are: (1) Abnormal rigidity of the spine, interfering with the passage of the trocar into the inter-laminar space; (2) blocking of the cannula by flakes of lymph or floating nerve-roots in the interior of the theca; (3) escape of pure blood by tapping a large spinal vein; (4) localised jerkings due to stimulation of a nerve root. With regard to the important question of the risk of the operation, he says: "It may legitimately be urged that any operative procedure which is essentially diagnostic in its aim must justify itself by showing a certain immunity from risk. Considering the large number of reported punctures, the gravity of the conditions for which they have been employed, and the extreme illness of many of the patients at the time, the mishaps have been comparatively few."

The procedure is considered from the two aspects —(1) Its diagnostic and (2) its therapeutic value. As a means of diagnosis it has proved useful in various conditions presenting the clinical evidences of increased intra-cranial tension—a class of cases in which differential diagnosis is often exceedingly difficult. Thus, in tubercular meningitis the detection of the tubercle bacillus has removed all doubt in a large number of cases. In epidemic cerebro-spinal meningitis, the diplococcus intercellularis and Fränkel's pneumococcus have been discovered. In purulent meningitis staphylococci have been found. The injection of the fluid withdrawn from a case of human rabies produced furious rabies in a dog and also in a rabbit. Sterile fluid was withdrawn from a case of acute mania. In addition to the detection of microorganisms in the fluid withdrawn, there are other points of diagnostic value, such as the presence of pus, or blood, the quantity of albumen—which is increased in acute inflammatory conditions to about 2 per cent. In chronic conditions, on the other hand, the proportion is about 0·4 to 0·8 per cent. A reducing substance of the nature of sugar is also present, but its diagnostic value is slight.

In examining the fluid, the quantity, pressure, specific gravity, colour and coagulability should be noted, as these characters vary in different diseases. The therapeutic value of the puncture is slight compared with its diagnostic value. Benefit, more or less transient, has followed in cerebral tumour, chronic hydrocephalus, uræmia, and spinal hæmorrhage; but no real advantage has been gained in cases of tubercular-meningitis, or rabies. Recovery has followed the operation in cases of acute serous or sero-purulent meningitis, chronic serous meningitis. and epidemic cerebro-spinal meningitis. Perhaps the most satisfactory therapeutic results of all have been obtained in the treatment of the severe and persistent headache associated with chlorosis.

Recent experimental work bearing upon lumbar puncture has been published by Wentworth, on the characters of the cerebro-spinal fluid. His conclusions are (1) that normal cerebro-spinal fluid contains neither cells nor fibrin, and is perfectly clear. (2) In cases of meningitis the cerebro - spinal fluid is invariably cloudy when withdrawn. The degree of cloudiness is to some extent proportionate to the amount and character of the exudation in the meninges. (3) The cloudiness is caused by cells; the character of the cells differs with the variety of the meningitis. After withdrawal fibrin is formed in the fluid. The presence of cells and fibrin is pathognomonic of meningitis. (4) The cloudiness is often exceedingly slight, and close observation is necessary to detect it. (5) In the normal fluid a trace of albumin is usually present, $\frac{1}{50}$th of one per cent. or less. In meningitis the percentage is increased. (6) The degree of force with which the fluid escapes from the needle has little diagnostic value as indicating an increased amount of fluid. (7) In a certain number of cases numerous white particles were present in the fluid when it was withdrawn. They were neither cells nor fibrin, and showed a tendency to dissolve after a time. They have nothing to do with meningitis, and must not be mistaken for cloudiness.[3]

The results of lumbar puncture in 19 cases in asylum practice are reported by Babcock. Twelve of the cases were general paralysis, two simple melancholia, one each of locomotor ataxia, stuperous melancholia, organic dementia, and status epilepticus. The therapeutic results were unsatisfactory. In only two cases was there any improvement at all, and that was slight.[4]

**Vertebral Osteomyelitis.**—Osteomyelitis of the vertebræ is a comparatively rare condition, and has only been clearly recognised as a distinct pathological entity within recent years. Chipault[5] contributes a paper, in which he discusses 31 cases collected from surgical literature. Three of these have been under his own observation, and his paper contains the clinical notes of these, as well as summaries of most of the other published cases, thus forming a valuable *résumé* of the subject.

It is interesting to note that the disease appears much more frequently in males than in females. Nineteen out of 23 cases in which the sex is mentioned were males. The disease seldoms manifests itself after the skeleton has ceased to grow, hence most of the patients are below 20 years of age. It occurs as early as six months, but is most common between 15 and 20. Sometimes the vertebræ are the primary and sole foci of disease; at other times other bones are infected. Staphlyococcus aureus and streptococcus pyogenes have been found in the pus. Trauma, exposure to cold, and fatigue have been apparent exciting causes in different cases. The pathological anatomy of vertebral osteomyelitis is the same as that of osteomyelitis elsewhere.

From an examination of the cases in which the level of the affected vertebræ has been noted, we find that 4 cases were in the cervical region, 5 in the dorsal, 14 in the lumbar, and 2 in the sacral. The arches and transverse processes appear to be affected more frequently than the bodies of the vertebræ, but sometimes the whole bone is involved. Spinal curvature due to giving way of the bodies is rare. The most important local complication resulting from this disease is the formation of abscesses in the vicinity. The seat of such purulent collections depends, of course, upon the part of the vertebra involved. So long as the arches alone are the seat of disease the resulting abscess shows posteriorly either in the middle line or laterally to the spine. If, on the other hand, the bodies are primarily infected, depending upon the level, retropharyngeal, mediastinal, psoas, or pelvic abscess will result. The main point of difference between these collections and those following on Pott's disease is the rapidity of their formation. In some cases the costo-vertebræ and sacro-iliac articulations have become implicated, and in others peri-dural collections of pus have been formed in the neural canal. The spinal and even the cerebral meninges have been secondarily infected in a few instances. In two cases œdematous myelitis resulted.

The symptoms are those of acute osteomyelitis in general, and the local manifestations are similar in kind to those of the same disease in long bones. Any complication present will, of course, give rise to corresponding signs and symptoms. The onset is sudden and the disease runs a rapid course, the patient often being in a serious condition within a few hours of the first evidence of illness.

The main source of difficulty in the diagnosis is the presence, early in the case, of symptoms of meningo-myelitis, when the primary disease is apt to be overlooked.

Surgical intervention, prompt and thorough, is the only rational line of treatment. Free incisions, extending if necessary into the dural space, and as free drainage being the main indications.

Results: The disease is fatal, if left alone, in the majority of cases. Of 16 cases treated surgically eight have recovered. Chipault has a patient who is well and sound five and a half years after operation, and he reasonably considers the patient permanently cured.

[1] The Hospital, Oct. 5, 1895; Mar. 9 and Nov. 7, 1896. [2] Scottish Med. and Surg. Jour., Feb., 1897. [3] Archives of Pediatrics, 1896. [4] State Hosp. Bulletin, New York, 1896. [5] Gaz. des Hospitaux, Dec. 12, 1896.

